# Long-term safety of gamma knife radiosurgery (SRS) for acromegaly

**DOI:** 10.1007/s11102-021-01149-0

**Published:** 2021-05-26

**Authors:** Hugh P. Sims‐Williams, Kaveesha Rajapaksa, John Yianni, Lee Walton, Saurabh Sinha, Matthias Radatz, Esther Herbert, Mike Bradburn, John Newell‐Price

**Affiliations:** 1 Department of Neurosurgery, Royal Hallamshire Hospital, Sheffield Teaching Hospitals NHS Foundation Trust, Sheffield, UK; 2 National Centre for Stereotactic Radiosurgery (SRS), Sheffield Teaching Hospitals NHS Foundation Trust, Sheffield, UK; 3 Department of Oncology and Metabolism, Medical School, University of Sheffield, Sheffield, UK; 4 Department of Endocrinology, Royal Hallamshire Hospital, Sheffield Teaching Hospitals NHS Foundation Trust, Sheffield, UK; 5 Clinical Trials Research Unit, School of Health and Related Research, University of Sheffield, Sheffield, UK

**Keywords:** Acromegaly, Hypopituitarism, Pituitary adenoma, Radiation, Radiosurgery, Fractionated radiotherapy, Stroke, Transient ischaemic attacks, Morbidity, Mortality, Complications, Safety

## Abstract

**Purpose:**

Acromegaly has high morbidity and mortality when growth hormone secretion remains uncontrolled. Stereotactic radiosurgery (SRS) may be used when pituitary surgery is not suitable or unsuccessful, but there are few very long-term safety data available, especially for significant adverse events such as stroke.

**Methods:**

118 patients with acromegaly were treated with SRS between 1985 and 2015, at the National Centre for Stereotactic Radiosurgery, Sheffield, UK. Data were gathered from case notes, hospital databases, and patient questionnaires. Stroke incidence in comparison to the normal population was quantified using the standardised incidence ratio (SIR), and visual complications assessed.

**Results:**

88% (104/118) had complete morbidity follow up data for analysis. The mean follow-up was 134 months, and median SRS dose was 30 Gy. 81% of tumours had cavernous sinus invasion. There was no excess stroke rate relative to that seen in two age- and sex-matched large population studies (SIR = 1.36, 95% CI 0.27–3.96; SIR = 0.52, 95% CI 0.06–1.89). In 68/104 patients who had MRI-guided SRS with no further radiation treatment (SRS or fractionated radiotherapy) there was no loss of visual acuity and 3% developed ophthalmoplegia. There was a positive correlation between > 1 radiation treatment and both ophthalmoplegia and worsening visual acuity.

**Conclusion:**

Stroke rate is not increased by SRS for acromegaly. Accurate MRI-based treatment planning and single SRS treatment allow the lowest complication rates. More than one radiation treatment (SRS or fractionated radiotherapy) was associated with increased visual complications.

**Supplementary Information:**

The online version contains supplementary material available at 10.1007/s11102-021-01149-0.

## Introduction

Uncontrolled acromegaly results in a three-fold increase in all-cause mortality which is reversible with normalisation of GH and IGH-1 levels [1–3]. Untreated acromegaly is associated with diabetes mellitus, hypertension, cardiomyopathy, obstructive sleep apnoea and increased risk of colorectal carcinoma [2]. Pituitary surgery is the recommended first line treatment for acromegaly [3, 4] and achieves immediate tumour and biochemical control in 65% of patients (45–64% of patients with macroadenomas and in 72–90% with microadenomas) [3–6]. Where surgery does not achieve complete remission or is contra-indicated, primary medical management can achieve biochemical control in up to 50% [3, 6]. In cases of inadequate control or poorly tolerated side effects using medication, fractionated radiotherapy (RT) (multiple low radiation doses over several weeks) or stereotactic radiosurgery (SRS) (high dose radiation using multiple axes of delivery) are established treatments that control tumour size and GH secretion [3, 4, 7]. Radiotherapy is, however, an independent risk factor for increased mortality [5, 8]. This increased mortality rate may not apply to SRS [3].

For acromegaly, SRS is the recommended form of radiation [3, 4, 9]. SRS offers endocrine remission in 40–60% of patients at 5–10 years and control of tumour growth in 93–100% [10]. SRS is thought to offer a more favourable side effect profile than RT [3, 9, 11], by minimising the radiation dose to normal tissue. The lack of effect on normal tissues is illustrated by the fact that in contrast to RT, SRS does not result in increased risk of secondary brain tumours [12, 13], but other non-endocrine complications including ophthalmoplegia, visual loss and trigeminal nerve dysfunction appear to relate to the region treated [9, 14, 15].

One specific concern about all forms of cranial radiation is the potential for long term cerebrovascular damage and stroke. Data on stroke following RT for pituitary tumours suggest an increased risk in some [1, 5, 16, 17], but not all series [12, 18, 19]. At the National Centre for Stereotactic Radiosurgery, Sheffield, UK we have used the gamma knife to deliver SRS to over 360 patients with pituitary disease over the last 35 years. Here, we present unique long-term data on 118 patients with acromegaly to assess stroke risk and safety of this treatment.

## Methods

### Radiosurgical technique

The use of the Leksell gamma knife (Elekta, Sweden) for the treatment of tumours has been well described [20]. 13 early patients were localised with CT, but since 1993 the subsequent 91 treatments were all based upon gadolinium enhanced T1 weighted volumetric MRI scans and planned with multiple isocentres mean 7 (range 1–21) using GammaPlan (Elekta AB, Sweden) (Table 1). Prior to 2011 cases were treated on the Gammaknife Model C following which all cases were treated on the Gammaknife Perfexion model. Median and mean isocontour of 50% (range 45–60%) was matched to the margin of the tumour and used to deliver a mean marginal dose of 28.0 Gy (Gy) (range 17.2–38.0) whilst maintaining a dose of ≤ 8 Gy to the optic apparatus. A maximum dose threshold was not applied to the cavernous sinus as the majority of tumours requiring treatment were within this area; opthalmoplegia incidence from SRS in our centre is low [21]. The mean treatment volume was 1949 cm^3^ (range 88–3938).

**Table 1:** Comparison of patient characteristics between those included in follow-up and those excluded due to inadequate data

Characteristic	Cohort	Lost to follow-up
Sample size (n)	104	14
Male:female	55:49	10:4
Median Follow up in months (range)	107 (18–362)	–
Median age at SRS	45	46
Median surgeries @ time of SRS (range)	1 (0–3)	1 (0–3)
Median (range) marginal dose	30 Gy (17.2–38)	25 Gy (20–35)
Mean (median) tumour volume (cm^3^)	1934 (972)*	1776 (985)
Mean (median) treatment volume (cm^3^)	1949 (1100)	2002 (1015)
Planning modality (MRI:CT)	91:13	13:1
Cavernous sinus invasion (%)	81% (84/104)	93% (13/14)
Fractionated radiotherapy (RT) prior to SRS	25% (26/104)	36% (5/14 patients in whom data available)
Co-morbidities at time of SRS		
ASA 3 or 4	20.2% (21/104)	21.4% (3/14)
Diabetes mellitus	10.6% (11/104)	14.3% (2/14)
Sleep apnoea	5.8% (6/104)	14.3% (2/14)
Valvular heart disease	3.9% (4/104)	0%
HTN	9.6% (10/104)	14.3% (2/14)
^#^Q-stroke score (10 year risk) Mean (Median)	2.5% (0.8%)	2.2% (0.8%)
Deaths (median age at death in years)	13 (67)	5 (62)
Median time to death from SRS in month (range)	102 (18–243)	–
Cause of death	Cardiac (3)	Unavailable
	- Tachyarrhythmia	
	- Left ventricular failure	
	- Acute pulmonary oedema secondary to AF	
	Respiratory(2)	
	- Idiopathic pulmonary fibrosis (asbestos)	
	- COPD	
	Infection (5)	
	- Pneumonia × 4	
	- Staphylococcus septicaemia	
	Malignancy (3)	
	- Oesophageal carcinoma	
	- Metastatic adenocarcinoma	
	- Metastatic cancer (unknown primary)	

*Data for two tumour volumes were estimated from their known treatment volume

^#^Qscores provided for 113 of 118 patients; unable to perform on patients aged less than 25 [25]

### Data collection

We performed a retrospective review of 118 patients undergoing gamma knife treatment for acromegaly between 1985 and 2015 at the National Centre for Stereotactic Radiosurgery, Sheffield, UK. In our role as national centre, patients are followed up for biochemical remission and complications by their local endocrinology service. Biochemical testing was performed at regional testing centres and results were not available to the authors of this study. At the time of referral patients had uncontrolled GH levels as determined by their regional endocrinology service. They were then considered by the Sheffield SRS-Pituitary MDT prior to being counselled with regard to their treatment options. Given the variation in assays, normal ranges and criteria for control over this long time period, it is not possible to quantify comparative disease burden at the time of treatment. Our study focussed on associated mortality and morbidity from SRS. Complication data however was available from clinic letters and via patient outcome survey to ensure quality of service provision. To mitigate against incongruent or missing clinical information, patients and general (family) practioners (GPs) were contacted directly to confirm or fill gaps of knowledge using a patient specific questionnaire (see appendix II). Pituitary function was inferred from the use of pituitary replacement therapy as directed by regional endocrinologists. Premorbid state of patients undergoing SRS was obtained from clinic letters or pre-SRS clinical assessment. Mortality was confirmed on The National Health Service (NHS) patient database and death certificates reviewed to find causality relating to SRS.

### Statistical methods

Overall survival was calculated as the time elapsed between first SRS treatment and death. Where death had not occurred, patients were censored at the last follow-up review (data collection completed in April 2018).

Due to retrospective data collection the exact date of new morbidity was not always available. Rules for generating an exact date for complication onset were applied uniformly.

The incidence of first stroke in comparison to the normal population was quantified using age-and-sex-matched incidence data. The incidence of strokes observed among patients in the gamma knife cohort was compared against two large population studies:

1. The Oxford Vascular study assessed stroke incidence in 90,000 people, selected by registration with a family physician in the defined area. The sample was 91% white and represented a higher than average median economic status; as our cohort are also from the UK, this was our primary reference population [22].
2. The City of Copenhagen population registry reported stroke incidence from a random sample of 19,698 patients taken from a population of 87,172 people, all of whom were older than 20 years; this was used to check the sensitivity of findings to a different reference population [23]. The City of Copenhagen study did not include transient ischaemic attacks (TIAs) in its definition of stroke and instances of TIA were removed from this comparison.

Both studies reported first stroke incidence by sex and 10-year age strata, enabling us to calculate the expected number of strokes over each patient’s follow-up time more accurately than overall population figures (see appendix for details). Person-years were defined as the duration from the date of first SRS to the date of stroke, date of death or date of record retrieval. 95% confidence intervals for the SIR were derived as described in Breslow and Day [24]. Factors associated with speed to remission, development of hypopituitarism, cerebrovascular events and other known complications were assessed by univariate analysis. Kaplan–Meier plots were performed to demonstrate the temporal association of developing hypocortisolism, hypogonadism and hypothyroidism. Those who were pan-hypopituitary pre-SRS were excluded from this specific analysis. A separate analysis was performed for those who had intact pituitary function pre-SRS. Two-sided Fisher’s exact tests were used to assess associations or trends where there were low expected complication rates.

## Results

### Patient characteristics

**Fig. 1:**
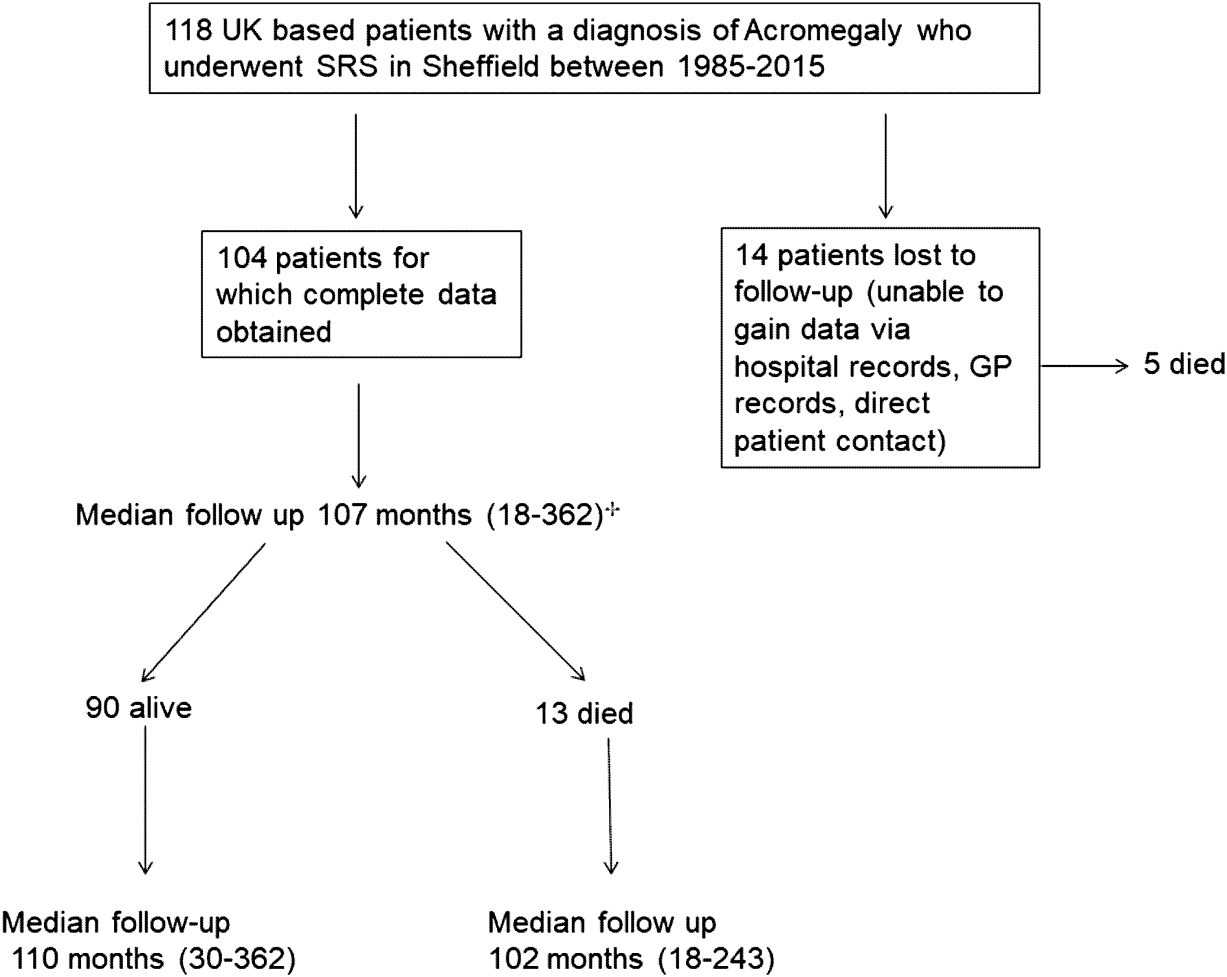
Flow diagram of included patients in study. ^+^In the MRI guided subgroup (n = 91) the mean follow up was 117 (median 96) months from SRS treatment

**Fig. 2:**
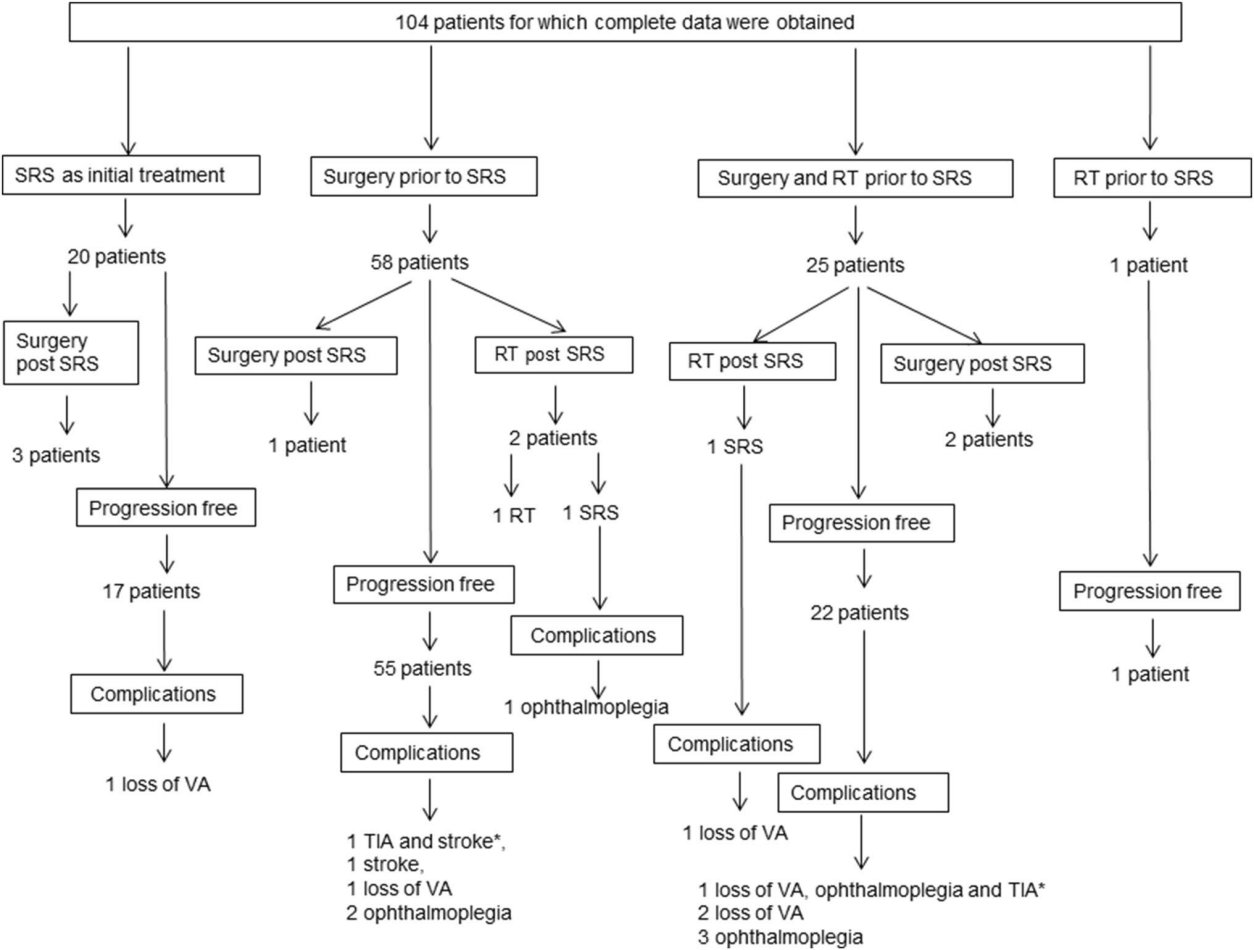
Summary of differing treatments received by the patients, pre and post stereotactic radiosurgery (SRS), and their relevant non-endocrine morbidity. *Indicates multiple morbidities occurred in a single patient

118 patients were treated using the gamma knife for acromegaly between 1985 and 2015, of whom detailed medical records were obtained for 104; a comparison of those with and without full data is provided in Fig. 1 and Table 1. Mean follow up was 134 months (median 107; range 18–362). In the MRI guided subgroup (n = 91) the mean follow up was 117 (median 96) months from SRS treatment. The number of patients not prescribed medication to control GH at last follow up halved relative to pre-SRS prescriptions (Supplementary table IIa) and the full range of therapies to control GH also reduced (Supplementary table IIb). Due to the complexity of treatment pathways a summary of non-endocrine morbidity as they relate to prior treatment is demonstrated in a flow diagram (Fig. 2).

### Mortality

Cause of death was ascertained for 18 of the 19 patients who had died. Median time to death post SRS was 102 months (range 18–243). No death was related to SRS treatment, but all deaths were attributable in part to the underlying diagnosis of acromegaly (Table 1).

### Non-endocrine morbidity

#### Stroke

Our cohort had a mean QRisk score [25] (10 year risk of stroke) of 2.3%; and considerably higher than average rates of hypertension, diabetes mellitus and valvular heart disease. Despite this, there was no increased risk of stroke relative to population studies. In total there were two Transient Ischaemic Accidents (TIAs) and two strokes in 3 patients which occurred at 3.9 years, 6.4 years, 6.9 and 8.7 years after SRS. The three affected patients all had MRI-guided SRS; two had cavernous sinus invasion; one had prior fractionated radiotherapy (Table 2). The remaining 101 patients were followed up between 1.6 and 30.2 years (mean 11.3 years). The expected incidence of first stroke based on the Oxford cohort population figures [22] was 2.21, giving a SIR of 1.36 (95% CI 0.27 to 3.96); in absolute terms, an extra 0.7 strokes per 1000 person-years. By contrast, fewer strokes (excluding TIAs) than expected were observed when standardising against the Copenhagen cohort [23] (expected number 3.83, SIR = 0.52, 95% CI 0.06 to 1.89) (see Appendix).

**Table 2:** Summary of patients who developed non-endocrine morbidity after SRS

Deficit	Description of symptoms (where available)	Date of SRS	Onset post SRS (months)	Dose (Gy)	Treatment volume (cc)	Treatment planning	Number of Operations	Cavernous sinus involvement	Date of TSPS	Date of RT	Total radiation treatments
Loss of acuity	Rapid visual loss post SRS; Imaging showed lesion on optic nerve. This was biopsied and showed gliosis and well differentiated astrocytoma. Possible dual pathology	12 January 1987	8	25	493	CT	0	No	N/A	n/a	1
Loss of acuity	Visual deterioration 14 months after SRS. Registered as blind 27years post SRS	25 February 1987	14	20	851	CT	1	No	1987	n/a	1
Loss of acuity	Left eye vision has deteriorated (but still functional); cause and timescale unknown	27 February 2002	n/a	20	5600	MRI	1	Yes	21/07/1996	01 October 1996	2
Loss of acuity	Documentation in notes of visual deterioration; patient had died at time of study; no further information available	14 September 1987	5	25	851	CT	1	No	1983	05 April 1983	2
Loss of acuity	Right eye blindness	09/07/1993 & 26 May 2004	105	30	2700	CT + MRI guided	1	Yes (2004)	1989	1990	3
Loss of acuity	Progressive loss of vision started 1 year post and progressed for 10 years. On review by SRS team at Sheffield; clinic letters support a diagnosis of SRS induced optic nerve injury	10 August 2005	12	30	2300	MRI	2	Yes	1999—diagnosis + TSPS; 2000 TSPS	01 June 2000	2
Ophthalmoplegia	Right CN3 palsy	24 February 2010	6	30	710	MRI	1	Yes	09/09/1999	01 March 2000	2
Ophthalmoplegia	Double vision since 2^nd^ SRS	05/07/2006 & 16/1/13	1	30	677.9	MRI	1	Yes (2006)	10/11/2005	n/a	2
Ophthalmoplegia	Intermittent double vision since SRS	18 June 2012	n/a	20	5830	MRI	1	Yes	01/11/2011	n/a	1
Ophthalmoplegia	Left CN3 palsy	30 October 2013	26	30	12,960	MRI	3	Yes	2009, 2009, 2010 April	01 July 2010	2
Opthalmoplegia	n/a	10 August 2005	24	30	2300	MRI	2	Yes	1999 TSPS; 2000 TSPS	01 June 2000	2
Opthalmoplegia	Left CN3 palsy	27 November 2012	2	25	11,110	MRI	1	Yes	24/01/2012	n/a	1
Opthalmoplegia	Left CN3 palsy	06 November 2013	n/a	30	1890	MRI	1	Yes	01/12/2009	06/11/2010	2
Trigeminal neuralgia	TGN since gamma knife	10 August 2005	0.1 (4 days)	30	2300	MRI	2	Yes	1999—diagnosis + TSPS; 2000 TSPS	01 June 2000	2
CVA	Dysarthria and left sided weakness—anterior circulation stroke 2015	12 December 2008	83	30	555.9	MRI	1	No	01/11/2003	n/a	1
TIA	01/08/2012 (TIA—no sustained neurological deficit; had incompetent heart valves); subsequently put on Warfarin & underwent open heart surgery for valve	10 August 2005	84	30	2300	MRI	2	Yes	1999—diagnosis + TSPS; 2000 TSPS	01 June 2000	2
CVA	TIA 28/05/2009 + Stroke 16/03/2014 (anterior circulation / leg affected); smoker	29 June 2005	47	30	1500	MRI	1	Yes	2004	n/a	1
Need for post STS TSPS	VIRGIN SRS; TSPS 1987	16 February 1987	9	20	1500	CT	1	No (later Cavernous sinus was involved)	04 December 1987	n/a	1
Need for post SRS TSPS	VIRGIN SRS; TSPS 1993	23 March 1987	80	20	8510	CT	1	Yes	01 December 1993	n/a	1
Need for post SRS TSPS	TSPS 1999; SRS 2002; further TSPS 2004	31 July 2002	18	20	4900	MRI	2	Yes	22/3/1999 & 29/1/2004	01 November 1999	2
Need for post SRS TSPS	VIRGIN SRS; TSPS 2011	07 December 2009	18	35	1000	MRI	1	No	01 June 2011	n/a	1
Need for post SRS TSPS	TSPS 1986; SRS 1987; further TSPS 1991	30 March 1987	48	20	493	CT	2	Yes	1986, 1991	30 January 1987	2
Need for post SRS TSPS	TSPS 2009; SRS 2011; further TSPS 2012	01 June 2011	18	30	2700	MRI	2	Yes	07/2009, 12/2012	n/a	1
Need for post SRS Radiation	TSPS 2005; SRS 2006; SRS 2013	05 July 2006 & 16 January 2013	78	30 + 30	677.9	MRI	1	Yes (left 2006)	10/11/2005	n/a	2
Need for post SRS Radiation	TSPS 1989; SRS 1993; SRS 2004	09 July 1993 & 26 May 2004	130	30 + 30	2700	MRI	1	Yes (right 2004)	1989	1990	3
Need for post SRS Radiation	TSPS 2004; SRS 2005; RT 2010	27 April 2005	65	30	1000	MRI	1	Yes	01/09/2004	27 March 2010 (LINAC)	2

#### Visual and cavernous sinus-related morbidity

A total of 26 non-endocrine complications affecting 22 patients occurred, making an incidence per patient year of 0.002. Further reduction in complication rates occurred with transition to MRI-guided SRS (n = 91) (Tables 2 and 3).

**Table 3:** Analysis of non-endocrine morbidity of stereotactic radiosurgery (SRS)

Deficit	Complication rate for SRS^@^ (n = 104) % of patients sustaining specific complication (no. of events)	Median onset	Complication rate for MRI-guided SRS ± RT (n = 91) *(October 1993–2015)*	Complications for single MRI-guided SRS; no previous RT (n = 68) *(October 1993–2015)*	Factors associated with increased risk
Loss of visual acuity	5.8% (6)^##^	12 months (6–106)	2.2% (2)	0%	> 1 radiation treatment (p = 0.04)
Ophthalmoplegia	6.7% (7)^##^	24 months (2–78)	7.7% (7)	2.9% (2)	> 1 radiation treatment (p = 0.03)
Trigeminal Neuralgia	1% (1)	4 days	1.1% (1)	0	N/A
Stroke/TIA	2.9% (3)	83.5 months (47–105)	3.3% (3)	2.9% (2)	Similar incidence to that expected for age-sex matched population
Need for further intervention—Surgery	5.8% (6)	18 (8–68)	3.3% (3)	2.9% (2)	
Need for further intervention—Radiation	2.9% (3)	78 (59–131)	N/A	N/A	

^##^Of those receiving a single MRI-guided SRS treatment and no fractionated radiotherapy there were no cases of visual loss and there were 2 cases of ophthalmoplegia

^@^Incidence of any non-endocrine complication per patient year was 0.002 (26/13,937)

In 84/104 patients there was cavernous sinus invasion. Of these 84 there were seven cases of ophthalmoplegia; all cases had MRI-guided treatment, targeting of the cavernous sinus due to tumour invasion, and had had prior pituitary surgery. Two of 68 patients receiving only MRI-guided SRS, and no other pituitary radiation, developed opthalmoplegia. Univariate analysis found > 1 radiation treatment to be a significant association with the occurrence of ophthalmoplegia (p = 0.03; Fisher’s exact two-tailed) giving an Odds ratio of 6.6(95% CI 1.2–34).

In the 68 receiving a single MRI-guided SRS treatment and no other cranial radiotherapy, there were no cases of visual loss. Overall, there were six cases of reduced visual acuity. Of these, four had undergone CT-guided treatment (historical mode of targeting prior to MRI being available), and two had had MRI-guided treatment and more than one radiation treatment. In the CT-guided treatment group 31% (4/13) developed loss of acuity, whereas 2.1% (2/91) developed reduced acuity after MRI-guided SRS and other pituitary radiation. CT-guided treatment was significantly associated with loss of visual acuity (p = 0.008; Fisher’s exact test, two-tailed), odds ratio 17.6 (95% 3.5–96.1). Repeat radiation treatment (of any type) was associated with reduced visual acuity (p = 0.04; Fisher’s exact test, two-tailed) with odds ratio of 6.5 (95% 1.4–35.2).

Visual complications were also associated with the number of interventions (i.e. surgery/radiation) undertaken to achieve growth hormone control, irrespective of treatment modality (Supplementary table I; p = 0.0001). There was an isolated case of trigeminal neuralgia in a patient who had MRI-guided SRS five years after fractionated radiotherapy.

### Hypopituitarism

**Fig. 3:**
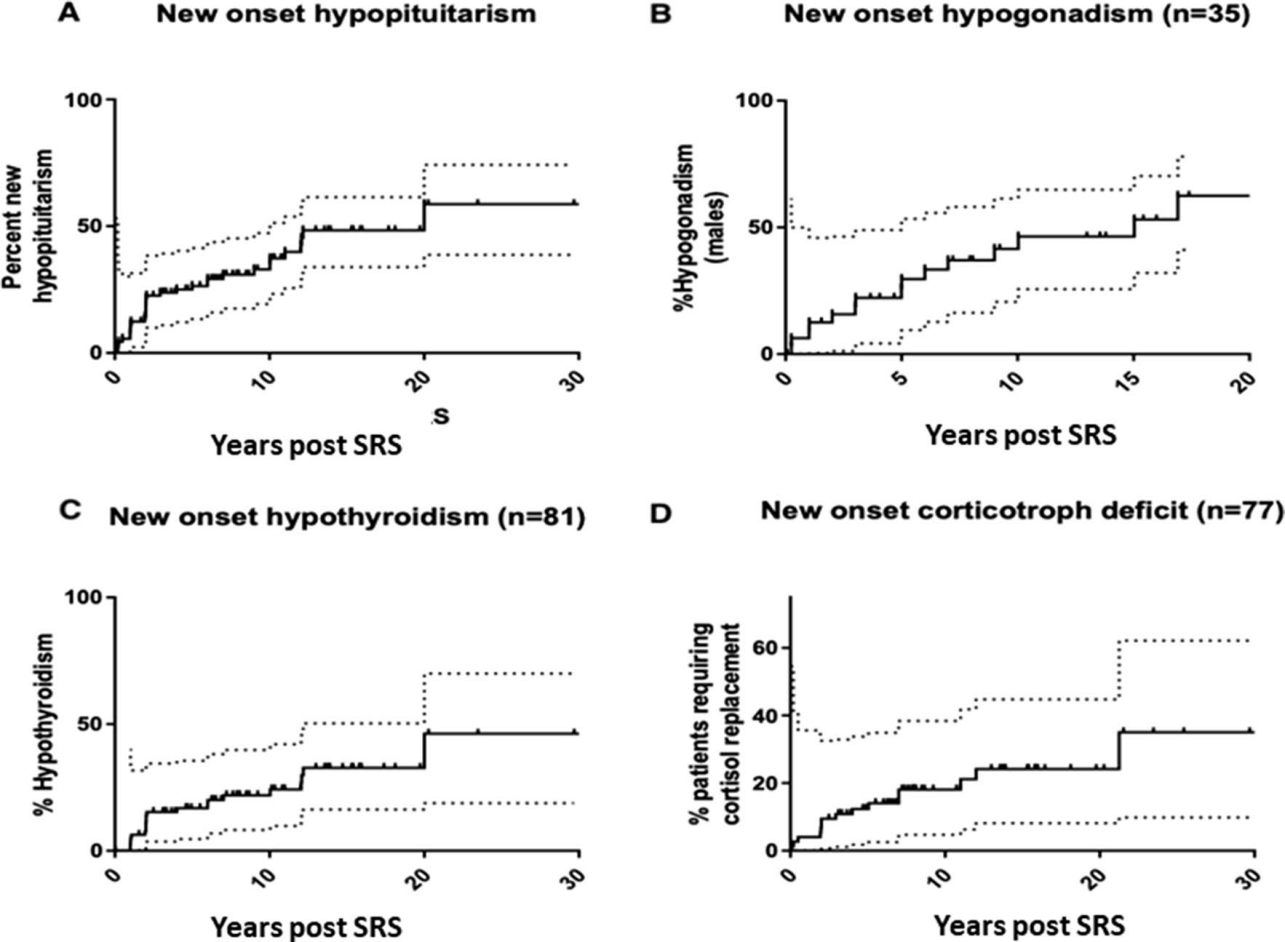
Onset of anterior pituitary axis deficit in our cohort over time. Seven patients had deficits in all 3 axes prior to stereotactic radiosurgery (SRS). Dotted lines represent 95% confidence intervals. **a** Incidence of hypopituitarism post SRS (excluding patients with panhypopituitarism pre-SRS) (34/97) **b** Incidence of testosterone replacement post SRS (16/35) **c** Incidence of thyroxine replacement therapy post-SRS (21/81) **d** Incidence of cortisol replacement therapy post-SRS (15/77)

Forty-three (41%) of the 104 patients had abnormal pituitary function prior to SRS treatment. Seven out of 104 patients required hormone replacement in 3 axes (thyroxine/cortisol/sex hormones) prior to treatment. In the remaining ninety-seven patients, 34 (38%) developed a new deficit in at least one axis after SRS. Of the 65 with normal pituitary function, 23 developed a new pituitary axis deficit (Supplementary table I). Overall, 46% (16/35) males with normal gonadal function pre-SRS developed hypogonadism, 26% (21/81) of euthyroid patients developed new thyroid deficiency and 19% (15/77) with normal corticotroph function developed new cortisol deficiency after SRS (Fig. 3). At 4 and 20 years, 25% and 50% of the cohort had developed a new axis deficit, respectively. The overall prevalence of pituitary dysfunction (all cause) in this cohort was 63% (66/104) at last follow up. Time for 25% of the population to develop hypogonadism (males only), hypothyroidism, and hypocortisolism was 5, 12, and 21 years, respectively. New onset of hypopituitarism occurred as late as 16-, 20-, and 21.2-years post SRS in the gonadal, thyrotroph, and corticotroph axes, respectively. There was an association between the severity (axes affected) and the number of interventions received (p = 0.007) (Supplementary Table I). A breakdown of pre and post SRS anterior pituitary deficits is provided in Supplementary Table II.

### Need for further intervention

Nine patients required further intervention post SRS, due to inadequate GH control (as determined by regional endocrinologists); three underwent fractionated radiotherapy, six underwent transsphenoidal surgery (Table 2).

## Discussion

We have demonstrated that SRS for acromegaly does not increase the risk of stroke when compared to control populations that were matched for both age and sex, and importantly by actual year of the event. To the best of our knowledge, such an analysis has not been done before for SRS to the pituitary region, where stroke risk due to disease is hard to decipher from treatment-related stroke risk. In other series, weighted mean risk of stroke in an acromegaly population post SRS is 0.3% (with a range across series of 0% to 5.7%) [10]. Of note the median dose was 21 Gy across these centres, lower than what we report here. In the same systematic review [10], the stroke rate for fractionated stereotactic radiotherapy was 4.5% (range across series 0% to 9%) where median dose was 49 Gy. Simple comparison of the stroke rate following SRS and RT is, however, confounded by the heterogenicity of treatment received and the background stroke risk of patients with uncontrolled growth hormone levels and the sequelae of acromegaly. There is likely both an observational and publication bias in the reporting of stroke in the literature. To avoid some of these confounders, our study sought to compare our SRS acromegaly cohort with that of similar age-matched populations by actual year. Whilst our data is specific for patients with acromegaly, we believe that it is likely that the observations will be generalisable to other patients with pituitary tumours.

Radiation therapy is thought to work not only by stopping tumour cell mitosis, but also through thrombo-obliterative and vascular changes. This mechanism explains its use in arteriovenous malformations and benign tumours, e.g. meningiomas [21]. Radiation can cause apoptosis and subsequent endothelial loss. Local inflammatory response and upregulation of hypoxia-related genes can lead to vascular injury, atherosclerosis and thromboembolism [26]. These data emphasise the potential issues pertaining to normal tissue receiving excess radiation and the need for our analyses here.

Historically, visual loss was observed where high doses reached the optic nerves [14, 15, 27]. Improvement in imaging quality and radiation delivery now ensures that the dose to the optic nerve can be kept less than 8 Gy, as recommended [4, 9]. Where tumour proximity would mean a higher dose being delivered to the optic nerve with SRS, surgery before SRS may be used to debulk the tumour and allow sufficient distance between tumour and optic apparatus for SRS to be safely administered, but if this is not possible fractionated radiation is preferred [4]. In our cohort, six patients developed loss of visual acuity at a median delay of 12 months; four of whom had > 1 radiation treatment, and four had now outdated CT-guided SRS; all patients had one or both of these risk factors (Table 2). In contrast there were no cases of visual loss in those who received only a single MRI-guided SRS treatment and no fractionated radiotherapy. Multiple treatments of any type, and regardless of order, appear to be associated with increased complication incidence (Supplementary table I). Rates of visual loss from SRS are reported to vary between centre, case selection and tumour location [10, 14, 15, 27]. It has been suggested that the tolerable absolute dose to the optic chiasm and volume treated varies between patients, as this may be altered by patient age, vascular comorbidities, previous treatments, and degree of nerve compression [10]. Nevertheless, it is not possible to offer firm guidance on dose reduction other than stating that the risk of visual loss appears greater with previous optic nerve irradiation and keeping the dose to the chiasm < 8 Gy appears to be safe for single treatments.

Ophthalmoplegia is a known complication of SRS when used on tumours invading the cavernous sinus. Literature reports incidence of new cranial neuropathies in either the oculomotor, trochlear, trigeminal nerves of 1.3% (21/1621), of which 50% were transient [28]. A more recent systematic review calculated a weighted mean for cranial nerve deficit of 0.45% (with rates per centre ranging from 0 to 5.7%) [10]. These are not seen where SRS dose is kept < 20 Gy [10] or where eloquent targets are kept below their threshold levels. Prior data from our unit has shown an ophthalmoplegia incidence of 1.1% for patients undergoing lower dose treatment (20 Gy) for skull-base meningiomas (of which 52% of tumours involved the cavernous sinus) [21]. The wide range of peripheral doses given (defined by the 50% isodose contour) doesn’t equate directly to dose reaching cranial nerves or pituitary gland, which is dependent on the precise dosimetry planning. Higher radiation doses are associated with more complications but the threshold of radiation that is associated with nerve damage is variable, with sensory nerves appearing to be more susceptible. In our acromegaly cohort, 81% (84/104) had cavernous sinus invasion, the mean dose was 28 Gy, and the incidence of ophthalmoplegia in those with cavernous sinus targeting was 8% (7/84). All seven cases of opthalmoplegia were MRI-guided, had had prior TSS, and had cavernous sinus invasion. Five of these seven had prior fractionated radiotherapy. Thus, ophthalmoplegia is a risk for patients undergoing SRS and who have cavernous sinus invasion, especially if there has been prior radiation therapy. Unfortunately, the method of data collection did not allow us to confirm whether the opthalmoplegia was transitory or permanent; other series’ suggest the half are transient. Careful counselling of patients is needed about this potential complication ahead of treatment.

Higher complication rates to vision and more severe hypopituitarism are seen in patients receiving two radiation treatments [27, 29]. It has been hypothesised that 40% [18]–50% [29] of the original radiation dose should be included in future dose calculations to the optic nerve. Our data demonstrate that where a single treatment of SRS is used (without concomitant fractionated radiotherapy), then lower complications rates to visual acuity and ophthalmoplegia are seen (p < 0.05) (Supplementary table I).

### Hypopituitarism

Hypopituitarism is a recognised side effect of RT, in the treatment of Acromegaly. Using fractionated radiotherapy, progressive hypopituitarism develops with 50–80% affected at 10 years mean follow-up [9]. Hypopituitarism of any type before treatment appears to increase the risk of subsequent hypopituitarism after fractionated radiotherapy [9]. Increased dose received by the pituitary stalk [30], cavernous sinus invasion [31], marginal (50%) dose (> 25 Gy) and tumour volume (> 2.5 ml) are associated with higher rates of hypopituitarism [14]. Our data found the total number of interventions of any type was also associated with degree of hypopituitarism (p < 0.01; Supplementary table I). Overall it appears that hypopituitarism levels are lower with SRS where rates of 10–50% at 5 years have been described [10, 11, 14]. However, the reported mean follow-up for those undergoing SRS is shorter than the follow-up period documented for fractionated radiation. The Virginia group published their SRS follow-up data (median 160 months; range 60–278 months) and demonstrated increased hypopituitarism with time [31]. In their cohort of 60 patients (24 Acromegaly: 36 Cushings Disease), the 10-year incidence of new hypopituitarism was 53.3% [31]. In our series 36% of our pre-SRS eupituitary cohort (n = 60) developed new hypopituitarism at median follow up of 114 months (mean 143). Our data suggests that SRS causes less hypopituitarism than fractionated radiation. However previous studies may have underreported the incidence of hypopituitarism, as it increases with length of follow-up. These data emphasise the need to long term follow-up and endocrine testing for this complication.

## Strengths and limitations

This is a large series with the longest recorded mean follow-up. We have carefully calculated the standardised incidence ratio (SIR) of stroke risk. Limitations include the retrospective nature of the study, reliance on recorded data and questionnaires, both of which introduce selection and recall bias. We are unable to report on growth hormone control due to the regional testing and management of Acromegaly in the UK. This is further limited by the regional variance seen in both testing methodology and frequency. We used active prescriptions for hormone replacement as a proxy for a biochemical diagnosis of hypopituitarism. While reflecting real-world medicine, our methodology may fail to capture sub-clinical or delayed hypopituitarism [32].

The design of this study and associated analyses are subject to confounding and our sample size was constrained by the number of patients having undergone SRS at our site. Nevertheless, the small number of SRS procedures undertaken and the long term follow-up means any prospective study would be a prohibitively long and expensive to address this question.

The Standardised Incidence Ratio (SIR) for cardiovascular accidents was derived by comparing the incidence against age/sex-matched data from two separate cohort studies, allowing us to demonstrate some robustness against the choice of reference population. Neither cohort contained details of other risk factors, and our SIR will be overestimated if patients undergoing SRS also have more risk factors which predispose to stroke (e.g. prior surgical procedures, co-morbidities). We are not aware of any data sets that contain extensive details on risk factors; moreover, if such nuanced data were to exist it would likely be far smaller than the two reference cohorts used here, meaning any gain in accuracy would come at a risk of a loss of precision. In absolute terms, the sparsity of stroke incidence (estimated as 3 per 1000 person-years) means it is unlikely the conclusions would change appreciably, were other reference populations used.

## Conclusion

This is the longest reported follow-up of any cohort of patients with acromegaly treated by gamma knife, specifically assessing morbidity and mortality. Our data show that there is no excess risk of stroke following SRS for acromegaly, and we believe that it is likely that this observation is generalisable to SRS for other pituitary tumours. Morbidity is associated with the number of interventions required to achieve GH control. When using MRI-planning and single SRS treatments, with no other form of radiation treatment, we found no risk for decreased visual acuity. Treating tumours in the cavernous sinus, especially where more than one radiation treatment is given, carries greater risk of opthalmoplegia and patients should be counselled about this potential effect. While the rate of hypopituitarism increases with time, it appears less prevalent than after fractionated radiotherapy. However patients having received SRS to the pituitary do need long term surveillance and testing for this.

## Supplementary Information

Below is the link to the electronic supplementary material.

Supplementary file1 (DOCX 20 kb)

Supplementary file2 (DOCX 19 kb)

## Abbreviations

AFL: Atrial fibrillation
ASA: The American Society of Anaesthesiologists Physical Status Score
CN3: Cranial nerve 3
COPD: Chronic Obstructive Pulmonary Disease
CT: Computed tomography
DoB: Date of birth
GH: Growth hormone
GP: General Physician
IGF-1: Insulin-like growth factor 1
MRI: Magnetic Resonance Imaging
N/A: Not available
RT: Fractionated radiotherapy
SRS: Gamma knife stereotactic radiosurgery
SIR: Standardised incidence ratio
TIA: Transient ischaemic attack
TSPS: Transsphenoidal pituitary surgery
VA: Visual acuity

## Declarations

### Public/patient consultation

Due to this being a quality improvement project, rather than research, there was no public and patient consultation regarding the key questions, study design etc.

### STROBE statement

It has been submitted separately.
