# Supplementary material for: Long-term safety of gamma knife radiosurgery (SRS) for acromegaly
Source: Pituitary. 2021 May 26;24(5):724–36. doi: 10.1007/s11102-021-01149-0 (PMC8416824; doi:10.1007/s11102-021-01149-0)
Supplement: Supplementary file 1 — Supplementary file1 (DOCX 20 kb) [file 11102_2021_1149_MOESM1_ESM.docx]

**Appendix I: Worked example of standardised incidence ratio (SIR)**

The calculation of the expected number of events is illustrated for two hypothetical patients using the OXVASC incidence figures. Patient A is male and undergoes SRS aged 55 years and 1 months and has a stroke aged 67 years and 3 months. The second is a female who undergoes SRS aged 62 years and 3 months and subsequently remains event-free until the age of 80 years and 9 months. The below details the total number of life-years spent in each risk strata and the cumulative expected number of events for each person. The denominator of the SIR is the expected number of events summed up across all patients.

| ***Age*** | ***<35*** | ***35-44*** | ***45-54*** | ***55-64*** | ***65-74*** | ***75-84*** | ***85+*** | ***Calculation*** |
| --- | --- | --- | --- | --- | --- | --- | --- | --- |
| **Males** |  |  |  |  |  |  |  |  |
| *Annual incidence per 1000 people^19^* | *0.00* | *0.27* | *0.73* | *1.77* | *6.46* | *9.42* | *19.72* |  |
| Patient A |  |  |  |  |  |  |  |  |
| Exposure time (person-years) | 0 | 0 | 0 | 9.92 | 2.25 | 0 | 0 | Observed = 1 |
| Cumulative risk (exposure time x risk) | - | - | - | 9.92x1.77  /1000  =0.0176 | 2.25x6.46  /1000  =0.0145 | - | - | Expected = 0.0321 |
| **Females** |  |  |  |  |  |  |  |  |
| *Annual incidence per 1000 people* | *0.00* | *0.16* | *0.54* | *1.75* | *4.08* | *10.51* | *15.08* |  |
|  |  |  |  |  |  |  |  |  |
| Patient 2  Exposure time (person-years) | *0* | *0* | *0* | *2.75* | *10* | *5.75* | *0* | Observed = 0 |
|  |  |  |  |  |  |  |  |  |
| Cumulative risk (exposure time x risk) | - | - | - | 2.75x1.75  /1000  =0.0048 | =10x4.08  /1000  =0.0408 | =5.75x10.51  /1000  =0.0604 | - | Expected =0.1060 |

**Appendix II:** Patient / General (family) Physician (GP) Questionnaire

The form was completed with the data we had and patients and GPs were required to fill in gaps or correct the information held and return the form by NHS fax.

| Name and DoB |  |
| --- | --- |
| Location for blood tests |  |
| Date(s) of pituitary surgery (if any) |  |
| Has the patient undergone fractionated radiation in addition to SRS? (date please) |  |
| Co-morbidities at time of Stereotactic radiosurgery (SRS) |  |
| Drug history of growth hormone suppressing medication starting from 6 months prior to SRS (Dopamine antagonists, Somatostatin analogues, pegvisommant) |  |
| Drug history for medication used in treatment of hypopituitarism (drug name and start date)  Eg. Thyroxine, Testosterone, Hydrocortisone, etc |  |
| Side effects of SRS if any? (and year developed)  I.e Ophthalmoplegia, visual loss, stroke, anterior skull base tumours |  |
| Cause & date of death if applicable |  |
