# Supplementary material for: Long-term safety of gamma knife radiosurgery (SRS) for acromegaly
Source: Pituitary. 2021 May 26;24(5):724–36. doi: 10.1007/s11102-021-01149-0 (PMC8416824; doi:10.1007/s11102-021-01149-0)
Supplement: Supplementary file 2 — Supplementary file2 (DOCX 19 kb) [file 11102_2021_1149_MOESM2_ESM.docx]

**Supplementary table I:** **The effect of multiple treatments on anterior pituitary function and non-endocrine morbidity.** Chi-Squared tests for trend performed; severity of pituitary dysfunction is associated with number of treatments (p = 0.007); visual complications are associated with number of treatments (p = 0.0001). This demonstrates association rather than causality. Treatments include gamma knife radiosurgery (SRS), transsphenoidal pituitary surgery or fractionated radiotherapy (RT) in any order.

**Legend for Supplementary table I:**

^#^ One female patient in this group required oestrogen replacement therapy.

^##^ Since our methodology was based solely on medication being required, we may underestimate the number of females with multiple-axis anterior pituitary deficits, due to the asymptomatic nature of an LH/FSH deficiency in a post-menopausal female. Within the dual-axis hypopituitarism group, 15 patients were female and may in fact have biochemically been demonstrating triple-axis hypopituitarism but not identified by our methodology.

^###^ All complications (events / no. of potential patients) from patients who underwent MRI-guided (stereotactic radiosurgery) SRS. Strokes were excluded as our data suggests this is not a complication of SRS. Hypopituitarism was also excluded as this is assessed elsewhere in the table.

**Supplementary Table I:**

|  | **No deficit** | **Single axis** | **Dual axis**^##^ | **Triple axis**^##^ | **% dual / triple axis hypopituitarism** | **Other complications ^###^** | **% visual complication rate ^###^** |
| --- | --- | --- | --- | --- | --- | --- | --- |
| **1 treatment (n=17)** | 7 | 5 | 1 | 4 | 29% (5/17) | none | 0% (0/17) |
| **2 treatments (n=54)** | 24 | 9 | 16 | 5^#^ | 39% (21/54) | 2 x ophthalmoplegia | 7.4% (2/54) |
| **3 treatments (n=23)** | 4 | 8 | 5 | 6 | 48% (11/23) | 3 x ophthalmoplegia  1 x loss of vision | 17% (4/23) |
| **4 treatments (n=8)** | 1 | 1 | 4 | 2 | 75% (6/8) | 1 x ophthalmoplegia, 2 x loss of vision | 50% (3/8) |
| **5 treatments (n=2)** | 0 | 0 | 1 | 1 | 100% (1/1) | 1 x ophthalmoplegia | 50% (1/2) |
| **Totals** | 37 | 23 | 26 | 18 |  | 10 |  |

**Supplementary table IIA:** Percentage of patients on medication pre SRS (<6months) and at last follow-up

|  | Pre SRS | At last follow up |
| --- | --- | --- |
| No medication | 25% | 53.9 % |
| On medication for GH control | 75% | 46.1% |

**Supplementary table IIB:** choice of medication prescribed

A summary of medications prescribed during the follow-up period against the medications being taken at last follow-up. Values given as frequencies rather than percentages as on occasion they were taken concomitantly.

|  | During treatment period | At last follow-up | % reduction |
| --- | --- | --- | --- |
| Dopamine Agonist | 34 | 17 | 50% |
| Somatostatin Analogue | 62 | 36 | 42% |
| Growth Hormone Receptor Antagonist | 10 | 5 | 50% |
